# Microarray-Based Comparisons of Ion Channel Expression Patterns: Human Keratinocytes to Reprogrammed hiPSCs to Differentiated Neuronal and Cardiac Progeny

**DOI:** 10.1155/2013/784629

**Published:** 2013-04-15

**Authors:** Leonhard Linta, Marianne Stockmann, Qiong Lin, André Lechel, Christian Proepper, Tobias M. Boeckers, Alexander Kleger, Stefan Liebau

**Affiliations:** ^1^Institute for Anatomy Cell Biology, Ulm University, Albert-Einstein Allee 11, 89081 Ulm, Germany; ^2^Institute for Biomedical Engineering, Department of Cell Biology, RWTH Aachen, Pauwelstrasse 30, 52074 Aachen, Germany; ^3^Department of Internal Medicine I, Ulm University, Albert-Einstein Allee 11, 89081 Ulm, Germany

## Abstract

Ion channels are involved in a large variety of cellular processes including stem cell differentiation. Numerous families of ion channels are present in the organism which can be distinguished by means of, for example, ion selectivity, gating mechanism, composition, or cell biological function. To characterize the distinct expression of this group of ion channels we have compared the mRNA expression levels of ion channel genes between human keratinocyte-derived induced pluripotent stem cells (hiPSCs) and their somatic cell source, keratinocytes from plucked human hair. This comparison revealed that 26% of the analyzed probes showed an upregulation of ion channels in hiPSCs while just 6% were downregulated. Additionally, iPSCs express a much higher number of ion channels compared to keratinocytes. Further, to narrow down specificity of ion channel expression in iPS cells we compared their expression patterns with differentiated progeny, namely, neurons and cardiomyocytes derived from iPS cells. To conclude, hiPSCs exhibit a very considerable and diverse ion channel expression pattern. Their detailed analysis could give an insight into their contribution to many cellular processes and even disease mechanisms.

## 1. Introduction

Ion channels are comprised of a large variety of differing families of pore proteins. Initially, ion channels were mostly known for their role in the nervous system where they play a crucial role in the signal transmission over neurites and synapses. But in fact they are involved in numerous other cellular processes including cell size regulation, muscle contractions, immune system activation [1], or hormone release [2]. Distinct ion channels are furthermore recognized to be of high importance for excitable cells of the heart: cardiomyocytes of the working myocardium as well as cells of the cardiac conduction system. In the heart, specific ion channels are responsible, for example, for the regulated generation of action potentials and for cardiac muscle contraction strength and time [3]. Additionally, ion channels play an important role in several differentiation and maturation processes [4–6]. The presented study aims to take a closer look at ion channel expression in human-induced pluripotent stem cells (hiPSCs) to give a start point for further analyses of their distinct roles at an early developmental cell state and differentiation processes. 

hiPSCs are generated from somatic cells by timed overexpression of specific transcription factors and strongly resemble pluripotent embryonic stem cells [7, 8]. Pluripotency is amongst others defined by the ability to differentiate into cells of all three germ layers and unlimited symmetrical cell division. This cell system is widely utilized for studies investigating developmental processes or disease mechanisms [9, 10]. Although it has become clear that differentiation processes influence ion channel expression [11], the distinct role of ion channels during these processes is so far only poorly understood. Nevertheless, it became evident that certain ion channels play a pivotal role in stem cell biology, including cell fate determination, cell cycle regulation, or cytoskeletal reorganization [6, 12–15].

The possibilities of iPSCs include the generation of individual or patient-specific-pluripotent cells, which can be subsequently differentiated into the affected cell types. This is already utilized to study pathomechanisms in a variety of tissues and cell types [9, 16–18]. The so-called channelopathies which are based on mutations in ion channels are the cause of developmental disorders and are the subject of various studies [19, 20]. To elucidate the role of ion channels in cell differentiation, maturation or their role in pathomechanisms a well-founded knowledge of ion channel distribution in pluripotent cells, representing one of the earliest stages of development, is indispensable. In that respect, we compared the expression levels of several ion channels in human keratinocytes with their reprogrammed progeny, hiPSCs. Keratinocytes derived from plucked human hair [21, 22] represent one of the most promising cell sources for the generation of the investigated hiPSCs [23]. We have elucidated ion channel regulation for various channel families and their subtypes. Additionally, we compared the expression levels of ion channel families and subtypes, found to be regulated during reprogramming, with iPSC-differentiated progeny, namely, neurons and cardiomyocytes. These comparisons could be a start point to evaluate the contribution and function of different ion channels, for example, for self-renewal and differentiation processes in stem cells. 

## 2. Material and Methods

### 2.1. Ethical Statement and Donor Information

After informed consent was given by the donors (ethical agreement by the University of Ulm, number 88/12) hair with intact hair roots was gathered by plucking from the scalp after desinfection. We used hair from healthy volunteers (age between 24 to 45 and both male and female gender).

### 2.2. hiPSC Generation and Cell Culture

Keratinocytes were obtained from plucked human scalp hair as already described [22]. Keratinocytes were propagated in EpiLife medium with HKGS supplement (both Invitrogen, Carlsbad, CA, USA). hiPSCs were generated from keratinocytes by lentiviral transduction of four reprogramming factors (Oct4, Sox2, Klf4, and cMyc) as described earlier [23]. After the reprogramming on rat embryonic fibroblasts they were maintained feeder-free on Matrigel (BD Biosciences, Franklin Lakes, NJ, USA) coated dishes in mTeSR1 medium (Stemcell Technologies, Vancouver, CA, USA).

### 2.3. Gene Expression Microarrays

Gene expression microarrays were performed for 6 keratinocyte samples and 9 hiPSC samples with the Agilent Whole Human Genome Microarray Kit (4x44k microarray kit G4112F, Agilent Technologies, Santa Clara, CA, USA). 500 ng of total RNA was used to produce Cy3-CTP-labeled cRNA with the Agilent Low RNA Input Liner Amplification Kit. The cRNA was purified and 1,65 *μ*g per array was hybridized for 17 h at 65°C and 10 r.p.m. Afterwards, the arrays were washed with Agilent Gene Expression Wash Buffers one and two and finally with acetonitrile for 1 min. The slides were scanned using Scan Control 7.0 software with a resolution of 5 *μ*m. Scan data was extracted with the Feature Extraction 9.1 software. Expression levels were background adjusted and quantile normalized with the GeneSpring GX 12 software. Differential expression between keratinocytes and hiPSCs was analyzed using student's *t*-test. A fold change > 2 and a *P* value < 0.05 was considered significant and highlighted bold (upregulation) or italic (downregulation) in the results table. For comparisons of iPSCs with iPSC-derived neurons published data from GSE34879 (GSM856936, GSM856937, GSM856915, GSM856916) and for cardiomyocytes GSE17579 (GSM438022, GSM438026, GSM438034, GSM438021, GSM438032, GSM438036) were used (both from NCBI Gene Expression Omnibus, http://www.ncbi.nlm.nih.gov/geo/). Fold change is shown if >2.

## 3. Results

### 3.1. Differential Expression of Ion Channels in hiPSCs Compared to Keratinocytes

We first compared the expression of various ion channel families from keratinocytes to hiPSCs and from hiPSCs to cardiomyocytes and neurons, respectively. From the 387 probes (Table 1) binding in ion channel genes from parental keratinocytes to hiPSCs, 101 (26%) showed a significant increase in expression (fold change > 2, *P* < 0.05; labeled in bold) while 23 (6%) showed a significant decrease (fold change > −2, *P* < 0.05; labeled in italic). In a second step differentially regulated ion channels from hiPSCs to hiPSC-derived neurons were investigated and we found 29 ion channel transcripts to be upregulated (fold change > 2; labeled in bold) while 6 showed a significant decrease (fold change > −2; labeled in italic). For cardiomyocytes, mRNA levels of only 7 ion channel members were upregulated (fold change > 2; labeled in bold) and 10 mRNA levels showed a significant decrease (fold change > −2; labeled in italic).

### 3.2. Voltage-Gated Calcium Channels

Voltage-gated calcium channels are crucially involved in the Ca^2+^-influx thereby playing an important role in calcium signaling of virtually all cells. High-voltage-gated calcium channels include the neural N-type channel, the poorly defined brain-specific R-type channel, the closely related P/Q-type channel, and the dihydropyridine-sensitive L-type channels responsible for excitation-contraction coupling of skeletal, smooth, and cardiac muscles as well as for hormone secretion in endocrine cells (reviewed in [24]). While mainly permeable for calcium ions they also show a low permeability for sodium ions. Upon depolarization of the cell they mediate a calcium influx into the cell. The channels consist of the main alpha subunit as well as regulatory beta, alpha 2/delta, and gamma subunits. The *α*_1_ subunit forms the ion conducting pore while the associated subunits have several functions including modulation of gating [25].* CACNA1A* mutations are for example involved in ataxia [26]. We have analyzed their expression with 42 probes. During reprogramming 14 (33%) of them are significantly upregulated while only 1 (2%) is significantly downregulated. From iPSCs to neurons, as expected, neuronal alpha-subunits *CACNA1D, CACNA1E *areupregulated together with several neuronal channel subunits (*α*_2_ *δ*, *β*, and *γ*). In cardiomyocytes, solely the alpha subunit of the cardiac *CACNA1C *is upregulated. Of note, none of the differentiated progeny downregulated any voltage-gated calcium channels.

### 3.3. Sperm-Associated Cation Channels

Sperm-associated cation channels or CatSper channels are calcium ion channels. They are flagellar proteins involved in sperm motility and therefore affect fertility [27]. During reprogramming to iPSCs, from seven used probes just one showed a significant downregulation and none had a significant upregulation. We observed nothing noteworthy in the differentiated progeny.

### 3.4. Nicotinic Acetylcholine Receptors

Nicotinic acetylcholine receptors play a role in interneuronal synapses and neuromuscular junctions. They are composed of five subunits as homomeric or heteromeric receptors. They are located at the postsynaptic site and upon binding of acetylcholine they allow the transmission of cations, especially sodium and potassium ions, in some versions also calcium ions. This leads to a depolarization of the membrane and triggers further signaling pathways [28]. Several acetylcholine receptors and subunits are thought to play roles in a variety of pathomechanisms, for example, psychiatric disorders, cardiovascular diseases, or cancer [29–31]. From 21 probes, 5 (24%) showed a significant upregulation while 3 (14%) showed a significant downregulation from keratinocytes to iPSCs. In neurons, solely the neuronal nicotinic acetylcholine receptor *CHRNA6* alpha subunit was upregulated, none in cardiomyocytes.

### 3.5. Cyclic Nucleotide-Gated Channels

Cyclic nucleotide-gated channels form tetrameric channels which—upon binding of cGMP—allow a flow of cations. For that, these channels track the intracellular concentration of cNMPs to produce a voltage response [32]. Their major role is the depolarization of rod photoreceptors, but they are also found in other tissues like olfactory sensory neurons [33], testis, kidney, or heart [34] and play a role in cellular development such as neuronal growth cone guidance [35]. Defects in these genes are reported to cause retinitis pigmentosa [36]. From 7 used probes one showed a significant upregulation and one showed a significant downregulation (both 14%) during reprogramming, while none was differentially regulated in differentiated neurons or cardiomyocytes.

### 3.6. GABA Receptors

GABA (gamma-aminobutyric acid) receptors are ligand-gated chloride channels. Since GABA is the main inhibitory neurotransmitter in the central nervous system GABA receptors play an important role for the brain function. The receptors are composed of five subunits which form heteromers. The GABA receptors are a drug target for anesthetics and other psychoactive drugs. We have analyzed 29 probes within GABA receptor subunits. From keratinocytes to iPSCs, 6 (21%) of them are significantly upregulated while just one (3%) is significantly downregulated. Interestingly, none was up- or downregulated in differentiated neurons, while *GABRB3* was downregulated and *GABRP* was upregulated in cardiomyocytes. Up to now, more or less nothing is known about GABA receptors or their subunits in cardiac cells. 

### 3.7. Glycine Receptors

Glycine receptors are inhibitory receptors of the postsynaptic site. They are activated by glycine and mediate an influx of chloride ions. Accordingly, GlyRs regulate not only the excitability of motor and sensory neurons but are also essential for the processing of photoreceptor signals, neuronal development, and inflammatory pain sensitization [37]. The heteromeric pore is formed by five subunits. Concerning their role in pathomechanisms, it was reported that mutations are causing hyperekplexia (also known as startle disease) [38]. We have analyzed 7 probes and none of them was significantly altered in hiPSCs. Transcript levels for the alpha2 subunit and the beta subunit were highly upregulated in differentiated neurons, pointing to their functional role in the nervous system.

### 3.8. Ionotropic Glutamate Receptors

Glutamate is the predominant excitatory neurotransmitter in the central nervous system. Therefore, ionotropic glutamate receptors play a key role for learning and memory processes. They are located in the postsynaptic membrane and are composed of several heteromeric subunits. Ionotropic glutamate receptors are further divided into AMPA (alpha-amino-3-hydroxy-5-methyl-4-isoxazole propionate), NMDA (N-Methyl-D-aspartate) or kainate receptors depending on their sensitivity for the agonists. From 36 probes, 5 (14%) showed a significantly increased expression and 2 (6%) showed a decreased expression in iPSCs compared to keratinocytes. As expected, several subunits were upregulated in neurons (none downregulated). None was upregulated in cardiomyocytes, while the kainate subtype *GRIK5* was downregulated.

### 3.9. Hyperpolarization-Activated Cyclic Nucleotide-Gated Channels

Hyperpolarization-activated cyclic nucleotide-gated channels are homodimers or heterodimers and form a hyperpolarization-activated potassium channel. CNG channels display a very complex heteromeric structure with various subunits and domains that play a critical role in their function [39]. They contribute to pacemaker currents in the heart [40] but are also found in neurons [41]. We have analyzed 5 probes and 3 (60%) of them showed a significant upregulation while none was downregulated after reprogramming. In neurons the *HCN3* channel transcript was upregulated, which plays a role in several neuronal functions including excitability of basal ganglia output neurons [42]. Although *HCN4* for example plays a critical role in the conduction system of the heart, we did not observe a specific regulation in cardiomyocytes.

### 3.10. Serotonin Receptors

Serotonin (or 5-hydroxytryptamine) receptors are ligand-gated receptors mainly found presynaptically in neurons. The type 3 receptor is the only ion channel while the other serotonin receptors are G-protein-coupled receptors. It forms a heteropentameric pore which upon activation by serotonin allows the flow of sodium and potassium, leading to a depolarization. Serotonin receptors modulate neuronal function and are therefore involved in various brain functions. The analysis of 6 probes for type 3 serotonin receptors showed a significant upregulation of 2 (33%, both for *HTR3A*) probes while none was downregulated in hiPSCs. Although we expected serotonin receptors to be upregulated during neuronal differentiation, no subunit was upregulated in neurons or cardiomyocytes, while only the receptor transcript for *HTR3A *was downregulated in neurons.

### 3.11. Voltage-Gated Potassium Channels

Voltage-gated potassium channels are composed of a large group of subunits with different characteristics concerning for example their inactivation speed. Functional channels are formed by heterotetramers. The channels are highly specific for potassium with a low affinity for sodium or other cations. Voltage-gated potassium channels are responsible for the repolarization of excitable cells following the sodium-mediated excitation of an action potential and are therefore found in neurons and other cells displaying action potentials. We have analyzed 81 probes. From keratinocytes to hiPSCs, 25 (31%) were significantly upregulated while only 3 (4%) were significantly downregulated. In neurons, several subunits were upregulated including *KCNB1*, coding for Kv2.1 and *KCND2*, both are better known for their role in cardiac cell excitability as well as *KCNF1, KCNH8,* and *KCNIP1*, all known for their contribution in neuronal excitability [43]. It should be noted that *KCNT1* is actually sodium activated but is included in the alphabetical list for a better overview. Further, several “S” subunits were upregulated (*KCNS2* in neurons) or downregulated (*KCNS3* in neurons; KCNS1 and 3 in cardiomyocytes). These subunits are unable to form functional channels as homotetramers but instead heterotetramerize with other alpha-subunits to form conductive channels. These subunits are involved in modifying the channels response and conductivity [44]. Few is known about distinct roles in other tissues, but they were associated with for example, pain modulation [45] or airway responsiveness [46].

### 3.12. Inwardly Rectifying Potassium Channels

Inwardly rectifying potassium channels have a higher tendency to allow the flow of potassium ions into the cell rather than to the outside of the cell. Therefore, they play an important role in the maintenance of the resting membrane potential. Their activation is constitutive or controlled by ATP binding and G-proteins [47]. Functional channels are formed as homo- or heterotetramers. These channels can be found predominantly in neurons, cardiac myocytes, the pancreas, or the kidneys. From 20 analyzed probes in hiPSCs compared to keratinocytes, 8 (40%) showed a significant upregulation and 3 (15%) were significantly downregulated. The GIRK2 channel encoded by *KCNJ6* is upregulated in neurons playing multiple roles in various tissues including the pancreas and brain [48, 49] and is associated with epileptic seizures in mice lacking the gene [50]. On the other hand, Kir6.1, encoded by *KCNJ8,* is upregulated in cardiomyocytes and has been reported to be involved in the pathogenesis of cardiac arrest in the early repolarization syndrome [51].

### 3.13. Two-P Potassium Channels

Two-P potassium channels contain two pore-forming P domains. After dimerization they form an outward rectifying potassium channel. They can be found in several tissues and are activated by various chemical or physical means (TRAAK channels). We have analyzed 23 probes. 4 (17%) of them were significantly upregulated while 4 (17%) were significantly downregulated. In neurons *KCNK3*, vital for setting the resting membrane potential and primary target for volatile anesthetics [52] as well as *KCNK4, *which is mechanically gated and contributes to axonal pathfinding, growth cone motility, and neurite elongation, as well as possibly having a role in touch or pain detection [53, 54], were upregulated. *KCNK5* and 6 were downregulated. In cardiomyocytes, downregulation of *KCNK5* and 12 was observed.

### 3.14. Calcium-Activated Potassium Channels

Calcium-activated potassium channels are mostly activated by intracellular calcium; some family members are also voltage gated. The family consists of large, intermediate, and small conductance family members. Channels are formed by two units (*KCNM* family) or most commonly four units (*KCNN* family). They are involved in, for example, afterhyperpolarization following the action potential and are predominantly found in neurons. Additionally, they are known to play different roles in cellular mechanisms, including stem cell biology [4–6, 55]. We have analyzed 18 probes of which 9 (50%) were significantly upregulated and none was significantly downregulated after reprogramming. In neurons, solely *KCNN2* was downregulated, while in cardiomyocytes *KCNMA1*, encoding the large conductance BK-channel, involved in heart rate regulation [56], was upregulated. 

### 3.15. P2X Receptors

P2X receptors are receptors for extracellular ATP and upon activation open a channel for ions, predominantly calcium. The channel is formed by homo- or heterotrimers. They are found in several tissues, mainly in the nervous system and muscle tissue. They are involved in a range of physiological processes such as modulation of synaptic transmission, vascular tone, cardiac rhythm, and contractility and immune response [57–61]. In stem cells an influence of P2X receptors on embryonic stem cell proliferation was reported [62]. From the 9 analyzed probes 1 (11%) was significantly upregulated and none downregulated. None of these receptors/channels was regulated in differentiated progeny. 

### 3.16. Transient Receptor Potential Channels

Transient receptor potential channels (TRP channels) are nonselective cation channels. They show different preferences for cations, as well as different activation mechanisms and functions. TRP channels are broadly expressed throughout the organism and mediate multiple functions. These include amongst others sensor activity for a wide range of hypertrophic stimuli and mutations in *TRPM4* are now recognized as causes of human cardiac conduction disorders (reviewed in [63]). Furthermore, TRP channels are related to the onset or progression of several diseases, and defects in the genes encoding TRP channels (so-called “TRP channelopathies”) underlie certain neurodegenerative disorders due to their abnormal Ca^2+^ signaling properties (reviewed in [64]). Additionally, TRP channels influence stem cell differentiation and survival [65, 66] and are involved in neuronal-stem-cell-derived development [67]. We have analyzed 40 probes. After reprogramming, 11 (28%) of them were significantly upregulated and none was significantly downregulated. In neurons, none of these channels was noteworthily regulated while solely *TRPC3* was upregulated in cardiomyocytes, reported to be involved in conduction disturbances induced by adenosine receptor A1AR by enhanced Ca^2+^ entry through the *TRPC3* channel [68]. 

### 3.17. Voltage-Gated Sodium Channels

Voltage-gated sodium channels consist of a main alpha unit and some optional modulating or regulatory subunits. They are highly selective for sodium and are involved in a variety of cellular functions including action potential formation [69]. From 24 probes we have analyzed, 6 (25%) were significantly upregulated and two (8%) were significantly downregulated from keratinocytes to iPS cells. These channels seem to be expressed in different kinds of stem cells and during development [70–72]. It was further reported that, for example, *SCN5A*, highly upregulated in iPS cells, is involved in cancer stem cell invasion [73]. *SCN2A*, *SCN3A,* and *SCN3B *were upregulated in neurons, described to be involved in neuronal excitation and epilepsy pathogenesis [74]. Although voltage-gated sodium channels play multiple roles also in the cardiac system [70, 75, 76], we observed no changes of this channel family in cardiomyocytes.

### 3.18. Nonvoltage-Gated Sodium Channels

Epithelial nonvoltage-gated sodium channels are amiloride sensitive. They form heterotrimers and are involved in ion and fluid transport across epithelia in several organs. We have analyzed 6 probes and one (17%) of them was significantly upregulated while none was significantly downregulated. Interestingly, the upregulated *SCNN1A* in iPS cells was subsequently downregulated both in neurons and cardiomyocytes, pointing to a possible function in stem cells. Of note, it was shown already that repression of pluripotency by retinoic acid represses the *SCNN1A* gene together with several other pluripotency factors [77].

### 3.19. Two-Pore Channels

Two-pore channels are cation-selective ion channels activated by the second messenger nicotinic acid adenine dinucleotide phosphate (NAADP). Upon activation calcium is released from intracellular stores [78]. We have analyzed 5 probes and while none of them was significantly upregulated two (40%) were significantly downregulated from keratinocytes to iPS cells. None of these channels was regulated in neurons or cardiomyocytes.

### 3.20. Zinc-Activated Ligand-Gated Ion Channels

The zinc-activated ligand-gated ion channel is activated upon binding of zinc. Until now just one family member is known that is expressed in several tissues [79]. Its exact function is not known. The expression was not significantly altered as shown by one probe in all cells.

## 4. Discussion

Although ion channels are mainly known for their role in electrically excitable cells they can be found in almost all tissues and are additionally involved in various processes such as cell differentiation and maturation [3, 4, 6, 12, 24, 47, 70]. These large groups of channel proteins are still underestimated concerning their role during embryonic development and cell fate determination. One of the most interesting *in vitro* models for the elucidation of both developmental processes and disease-specific cellular impairments is represented by pluripotent stem cells. Therefore we were interested in the set of ion channels expressed in hiPSCs after reprogramming and compared this set with iPSC-derived differentiated progeny, namely, neurons and cardiomyocytes. As little is known about ion channels in hiPSCs we aimed to start the analysis with gene expression microarray data. For a comparison, we chose the cells from which they were produced—namely, keratinocytes—as the reference cell type. The comparison of 6 keratinocyte samples with 9 hiPSC samples should minimize the often observed variances between hiPSC lines. We found out that almost a third (32%) of the ion channel probes we investigated showed a significant change in gene expression. Of note, this was mostly an upregulation. Additionally, while many ion channel genes were not expressed in keratinocytes they were present in hiPSCs. This indicates that several of the analyzed channel groups might play unknown roles in stem cell biology, for example, homeostasis, proliferation, or differentiation. Interestingly, after differentiation into neurons or cardiomyocytes, relatively small groups were subsequently regulated. This includes ion channel transcripts playing important roles in the respective tissues. Nevertheless, we compared already published sets of data from different experimental setups and additionally limited the analysis to a strong fold regulation. This might lead to high dropout rates of regulated genes during measurement. Still, various channel transcripts were “logically” up- or downregulated during differentiation into neurons or cardiomyocytes, following embryonic development. iPS cells and especially patient-specific iPS cells from persons suffering from genetic mutations leading to hereditary syndromes are a very valuable tool to investigate pathogenetic mechanisms and disease associated molecular and cellular changes [80–82]. As various channel subtypes are involved in multiple pathogenetic mechanisms it would be further interesting to analyze channel transcript regulation in patient specific iPS cells and their differentiated progeny to elucidate possible disease-specific pathways. 

Concerning the presented study it is clear that gene regulations on transcript level do not explicitly mimic either protein levels and posttranslational modifications or protein activity. This set of data is sought to describe a global overview on transcript regulation of ion channels during distinct steps of development. It should be noted that in cases where several probes bind within one gene they do not indicate the same upregulation. Sometimes they show the same trend but miss significance, but for some cases there are considerable differences. This could hint for some yet unknown splicing variants. More detailed studies of these hypothesized splice variants could give insights into their function and broaden the still scarce knowledge.

This work is intended to be a guide and start point for future work focusing on single channels and their composition, localization, and function in hiPSCs and their differentiated progeny. These studies could lead to better *in vitro* differentiation protocols but also explain some of the many disease pathomechanisms related to mutations in ion channel genes.

## Figures and Tables

**Table 1a:** (a) Voltage-gated calcium channels

Gene name	Accession number	Gene symbol	*P*	Fold change Ker → hiPSC	Fold change hiPSC → Neuron	Fold change hiPSC → CM
Calcium channel, voltage-dependent, P/Q type, alpha 1A subunit, transcript variant 1	NM_000068	CACNA1A	2.26*E* − 01	−1.32		
Calcium channel, voltage-dependent, N type, alpha 1B subunit	NM_000718	CACNA1B	7.27*E* − 01	−1.10		
Calcium channel, voltage-dependent, N type, alpha 1B subunit [Source: HGNC Symbol; Acc: 1389]	ENST00000277550	CACNA1B	7.15*E* − 01	−1.14		
Calcium channel, voltage-dependent, N type, alpha 1B subunit	NM_000718	CACNA1B	6.32**E** − 09	**6.82**		
Calcium channel, voltage-dependent, N type, alpha 1B subunit [Source: HGNC Symbol; Acc: 1389]	ENST00000277551	CACNA1B	3.32*E* − 02	−1.57		
Calcium channel, voltage-dependent, L type, alpha 1C subunit, transcript variant 18	NM_000719	CACNA1C	6.77*E* − 02	−1.37		
Calcium channel, voltage-dependent, L type, alpha 1C subunit, transcript variant 18	NM_000719	CACNA1C	5.72**E** − 03	**2.37**		**2.94**
Calcium channel, voltage-dependent, L type, alpha 1D subunit, transcript variant 1	NM_000720	CACNA1D	3.83*E* − 01	1.58		
Calcium channel, voltage-dependent, R type, alpha 1E subunit, transcript variant 3	NM_000721	CACNA1E	*3.10 E*−*02 *	*−4.48 *	**2.08**	
Calcium channel, voltage-dependent, R type, alpha 1E subunit [Source: HGNC Symbol; Acc: 1392]	ENST00000524607	CACNA1E	8.83*E* − 01	1.05		
Calcium channel, voltage-dependent, L type, alpha 1F subunit	NM_005183	CACNA1F	8.92*E* − 01	1.02		
Calcium channel, voltage-dependent, T type, alpha 1G subunit, transcript variant 15	NM_198397	CACNA1G	6.42*E* − 01	−1.12	**4.60**	
Calcium channel, voltage-dependent, T type, alpha 1G subunit, transcript variant 1	NM_018896	CACNA1G	5.37**E** − 07	**6.96**		
Calcium channel, voltage-dependent, T type, alpha 1H subunit, transcript variant 1	NM_021098	CACNA1H	7.38**E** − 10	**6.78**		
Calcium channel, voltage-dependent, T type, alpha 1I subunit, transcript variant 1	NM_021096	CACNA1I	2.22**E** − 11	**37.25**		
Calcium channel, voltage-dependent, T type, alpha 1I subunit, transcript variant 1	NM_021096	CACNA1I	7.56*E* − 02	−1.56		
Calcium channel, voltage-dependent, L type, alpha 1S subunit	NM_000069	CACNA1S	5.22*E* − 02	−1.97		
Calcium channel, voltage-dependent, alpha 2/delta subunit 1	NM_000722	CACNA2D1	1.38**E** − 09	**9.65**		
Calcium channel, voltage-dependent, alpha 2/delta subunit 2, transcript variant 1	NM_001005505	CACNA2D2	3.30**E** − 10	**62.50**	**2.02**	
Calcium channel, voltage-dependent, alpha 2/delta subunit 3	NM_018398	CACNA2D3	5.14**E** − 05	**5.42**	**2.55**	
Calcium channel, voltage-dependent, alpha 2/delta subunit 3	AF516696	CACNA2D3	4.68**E** − 11	**33.67**		
Calcium channel, voltage-dependent, alpha 2/delta subunit 4	NM_172364	CACNA2D4	2.95*E* − 01	−1.41		
Calcium channel, voltage-dependent, beta 1 subunit, transcript variant 3	NM_199248	CACNB1	4.31*E* − 01	−1.14		
Calcium channel, voltage-dependent, beta 1 subunit, transcript variant 1	NM_000723	CACNB1	8.02*E* − 01	−1.02		
Calcium channel, voltage-dependent, beta 1 subunit, transcript variant 1	NM_000723	CACNB1	6.22*E* − 01	1.04		
cDNA FLJ45229 fis, clone BRCAN2020972,	AK128769	CACNB2	9.78*E* − 01	1.02		
Calcium channel, voltage-dependent, beta 2 subunit, transcript variant 1	NM_000724	CACNB2	4.71**E** − 02	**3.45**		
Calcium channel, voltage-dependent, beta 3 subunit, transcript variant 1	NM_000725	CACNB3	3.55*E* − 02	−1.93	**9.56**	
Calcium channel, voltage-dependent, beta 3 subunit, transcript variant 1	NM_000725	CACNB3	6.61*E* − 01	1.09		
Calcium channel, voltage-dependent, beta 4 subunit, transcript variant 1	NM_001005747	CACNB4	1.33**E** − 03	**4.43**		
Calcium channel, voltage-dependent, gamma subunit 1	NM_000727	CACNG1	9.46*E* − 01	1.02		
Calcium channel, voltage-dependent, gamma subunit 2	NM_006078	CACNG2	4.19*E* − 01	−1.29		
Calcium channel, voltage-dependent, gamma subunit 2	NM_006078	CACNG2	7.91*E* − 01	1.09		
Calcium channel, voltage-dependent, gamma subunit 3	NM_006539	CACNG3	2.21*E* − 01	−1.95		
Calcium channel, voltage-dependent, gamma subunit 4	NM_014405	CACNG4	4.93*E* − 01	1.14		
Calcium channel, voltage-dependent, gamma subunit 5	NM_145811	CACNG5	7.49*E* − 01	−1.13		
Calcium channel, voltage-dependent, gamma subunit 5 [Source: HGNC Symbol; Acc: 1409]	ENST00000169565	CACNG5	2.19*E* − 01	−1.56		
Calcium channel, voltage-dependent, gamma subunit 6, transcript variant 1	NM_145814	CACNG6	5.56**E** − 11	**46.40**		
Calcium channel, voltage-dependent, gamma subunit 7	NM_031896	CACNG7	3.54**E** − 13	**1439.36**	**3.37**	
Calcium channel, voltage-dependent, gamma subunit 7	NM_031896	CACNG7	1.82*E* − 01	1.26		
Calcium channel, voltage-dependent, gamma subunit 8	NM_031895	CACNG8	2.01*E* − 01	1.43		
Calcium channel, voltage-dependent, gamma subunit 8	NM_031895	CACNG8	1.18**E** − 09	**9.70**		

**Table 1b:** (b) Sperm-associated cation channels

Gene name	Accession number	Gene symbol	P	Fold change Ker → hiPSC	Fold change hiPSC → Neuron	Fold change hiPSC → CM
Cation channel, sperm associated 1	NM_053054	CATSPER1	3.26*E* − 01	1.51		
Cation channel, sperm associated 2, transcript variant 2	NM_172095	CATSPER2	*1.32 E*−*04 *	*−2.78 *		
Cation channel, sperm associated 2, transcript variant 4	NM_172097	CATSPER2	3.12*E* − 02	−1.78		
Cation channel, sperm associated 3	NM_178019	CATSPER3	1.50*E* − 03	1.93		
Cation channel, sperm associated 4	NM_198137	CATSPER4	2.51*E* − 01	1.48		
Cation channel, sperm-associated, beta	NM_024764	CATSPERB	4.38*E* − 01	−1.25		
Cation channel, sperm-associated, gamma	NM_021185	CATSPERG	3.56*E* − 01	1.34		

**Table 1c:** (c) Nicotinic acetylcholine receptors

Gene name	Accession number	Gene symbol	P	Fold change Ker → hiPSC	Fold change hiPSC → Neuron	Fold change hiPSC → CM
Cholinergic receptor, nicotinic, alpha 1 (muscle), transcript variant 1	NM_001039523	CHRNA1	8.06*E* − 01	−1.10		
Cholinergic receptor, nicotinic, alpha 1 (muscle), (cDNA clone IMAGE: 4124038), with apparent retained intron	BC006314	CHRNA1	4.64*E* − 01	1.47		
Cholinergic receptor, nicotinic, alpha 2 (neuronal)	NM_000742	CHRNA2	6.05*E* − 01	−1.30		
Cholinergic receptor, nicotinic, alpha 3, transcript variant 1	NM_000743	CHRNA3	1.84**E** − 10	**34.22**		
Cholinergic receptor, nicotinic, alpha 4	NM_000744	CHRNA4	6.79*E* − 01	1.15		
Cholinergic receptor, nicotinic, alpha 4 (neuronal), exon 1	X89741	CHRNA4	*2.79 E*−*02 *	*−2.53 *		
Cholinergic receptor, nicotinic, alpha 5	NM_000745	CHRNA5	4.03**E** − 05	**3.41**		
Cholinergic receptor, nicotinic, alpha 6, transcript variant 1	NM_004198	CHRNA6	2.23*E* − 01	−1.27	**2.21**	
Cholinergic receptor, nicotinic, alpha 7, transcript variant 1	NM_000746	CHRNA7	4.61*E* − 04	1.86		
Cholinergic receptor, nicotinic, alpha 7, transcript variant 2	NM_001190455	CHRNA7	1.56*E* − 11	13.24		
Cholinergic receptor, nicotinic, alpha 9	NM_017581	CHRNA9	6.30**E** − 06	**6.15**		
Cholinergic receptor, nicotinic, alpha 10	NM_020402	CHRNA10	1.57*E* − 02	1.57		
Cholinergic receptor, nicotinic, beta 1 (muscle)	NM_000747	CHRNB1	*8.07 E*−*04 *	*−2.47 *		
Cholinergic receptor, nicotinic, beta 1 (muscle)	NM_000747	CHRNB1	8.87*E* − 03	−1.92		
Cholinergic receptor, nicotinic, beta 2 (neuronal)	NM_000748	CHRNB2	*6.03 E*−*04 *	*−3.01 *		
Cholinergic receptor, nicotinic, beta 2 (neuronal) [Source: HGNC Symbol; Acc: 1962]	ENST00000368476	CHRNB2	2.98**E** − 09	**32.05**		
Cholinergic receptor, nicotinic, beta 3	NM_000749	CHRNB3	5.91*E* − 01	1.12		
Cholinergic receptor, nicotinic, beta 4	NM_000750	CHRNB4	1.76**E** − 02	**4.20**		
Cholinergic receptor, nicotinic, delta	NM_000751	CHRND	9.84*E* − 01	1.01		
Cholinergic receptor, nicotinic, epsilon	NM_000080	CHRNE	3.66*E* − 01	−1.20		
Cholinergic receptor, nicotinic, gamma	NM_005199	CHRNG	6.70*E* − 01	1.14		

**Table 1d:** (d) Cyclic nucleotide-gated channels

Gene name	Accession number	Gene symbol	P	Fold change Ker → hiPSC	Fold change hiPSC → Neuron	Fold change hiPSC → CM
Cyclic nucleotide-gated channel alpha 1, transcript variant 2	NM_000087	CNGA1	7.14*E* − 01	1.15		
Cyclic nucleotide-gated channel alpha 1, transcript variant 2	NM_000087	CNGA1	1.18**E** − 02	2.00		
Cyclic nucleotide-gated channel alpha 3, transcript variant 1	NM_001298	CNGA3	2.00*E* − 01	1.21		
Cyclic nucleotide-gated channel alpha 4	NM_001037329	CNGA4	2.78*E* − 01	1.82		
Cyclic nucleotide-gated channel beta 1, transcript variant 1	NM_001297	CNGB1	*9.22 E*−*03 *	*−2.05 *		
Cyclic nucleotide-gated channel beta 1, transcript variant 1	NM_001297	CNGB1	6.36*E* − 01	−1.13		
Cyclic nucleotide-gated channel beta 3	NM_019098	CNGB3	2.93*E* − 01	1.78		

**Table 1e:** (e) GABA receptors

Gene name	Accession number	Gene symbol	P	Fold change Ker → hiPSC	Fold change hiPSC → Neuron	Fold change hiPSC → CM
Gamma-aminobutyric acid (GABA) A receptor, alpha 1, transcript variant 1	NM_000806	GABRA1	1.26*E* − 01	1.41		
Gamma-aminobutyric acid (GABA) A receptor, alpha 1, transcript variant 1	NM_000806	GABRA1	8.87*E* − 01	1.05		
Gamma-aminobutyric acid (GABA) A receptor, alpha 2, transcript variant 1	NM_000807	GABRA2	9.70*E* − 02	1.70		
Gamma-aminobutyric acid (GABA) A receptor, alpha 2, transcript variant 1	NM_000807	GABRA2	4.49*E* − 01	1.48		
Gamma-aminobutyric acid (GABA) A receptor, alpha 3	NM_000808	GABRA3	2.10*E* − 01	1.43		
Gamma-aminobutyric acid (GABA) A receptor, alpha 4, transcript variant 1	NM_000809	GABRA4	3.76*E* − 01	1.43		
Gamma-aminobutyric acid (GABA) A receptor, alpha 4, transcript variant 1	NM_000809	GABRA4	8.30*E* − 01	1.05		
Gamma-aminobutyric acid (GABA) A receptor, alpha 5, transcript variant 1	NM_000810	GABRA5	1.09**E** − 08	**14.41**		
Gamma-aminobutyric acid (GABA) A receptor, alpha 6	NM_000811	GABRA6	1.69*E* − 01	1.23		
Gamma-aminobutyric acid (GABA) A receptor, beta 1	NM_000812	GABRB1	1.14*E* − 01	1.43		
Gamma-aminobutyric acid (GABA) A receptor, beta 2, transcript variant 2	NM_000813	GABRB2	1.27*E* − 02	1.94		
Gamma-aminobutyric acid (GABA) A receptor, beta 3, transcript variant 1	NM_000814	GABRB3	6.70**E** − 10	**85.93**		*−4.49 *
Gamma-aminobutyric acid (GABA) A receptor, beta 3, transcript variant 1	NM_000814	GABRB3	1.15**E** − 09	**115.69**		
Gamma-aminobutyric acid (GABA) A receptor, delta	NM_000815	GABRD	6.26*E* − 02	3.02		
Gamma-aminobutyric acid (GABA) A receptor, epsilon	NM_004961	GABRE	*5.83 E*−*05 *	*−5.47 *		
Gamma-aminobutyric acid (GABA) A receptor, Gamma 1	NM_173536	GABRG1	1.78*E* − 01	1.24		
Gamma-aminobutyric acid (GABA) A receptor, Gamma 1	NM_173536	GABRG1	1.70**E** − 02	**2.19**		
Gamma-aminobutyric acid (GABA) A receptor, Gamma 2, transcript variant 1	NM_198904	GABRG2	6.02*E* − 01	1.14		
Gamma-aminobutyric acid (GABA) A receptor, Gamma 2, transcript variant 2	NM_000816	GABRG2	5.28*E* − 02	1.72		
Gamma-aminobutyric acid (GABA) A receptor, Gamma 2, transcript variant 2	NM_000816	GABRG2	9.47**E** − 09	**8.41**		
Gamma-aminobutyric acid (GABA) A receptor, Gamma 3	NM_033223	GABRG3	1.99*E* − 01	2.00		
Gamma-aminobutyric acid (GABA) A receptor, Gamma 3	NM_033223	GABRG3	1.04*E* − 01	1.61		
Gamma-aminobutyric acid (GABA) A receptor, pi	NM_014211	GABRP	6.62*E* − 01	−1.12		**17.25**
Gamma-aminobutyric acid (GABA) receptor, theta	NM_018558	GABRQ	1.60*E* − 01	1.56		
Gamma-aminobutyric acid (GABA) receptor, theta	NM_018558	GABRQ	4.76*E* − 01	−1.27		
Gamma-aminobutyric acid (GABA) receptor, theta [Source: HGNC Symbol; Acc: 14454]	ENST00000370306	GABRQ	8.22**E** − 09	**52.17**		
Gamma-aminobutyric acid (GABA) receptor, rho 1	NM_002042	GABRR1	8.94*E* − 01	−1.04		
Gamma-aminobutyric acid (GABA) receptor, rho 2	NM_002043	GABRR2	2.83*E* − 01	1.54		
Gamma-aminobutyric acid (GABA) receptor, rho 3	NM_001105580	GABRR3	3.05*E* − 01	−1.34		

**Table 1f:** (f) Glycine receptors

Gene name	Accession number	Gene symbol	P	Fold change Ker → hiPSC	Fold change hiPSC → Neuron	Fold change hiPSC → CM
Glycine receptor, alpha 1, transcript variant 2	NM_000171	GLRA1	5.47*E* − 01	−1.29		
Glycine receptor, alpha 2, transcript variant 1	NM_002063	GLRA2	1.17*E* − 01	1.42	**11.41**	
Glycine receptor, alpha 3, transcript variant 1	NM_006529	GLRA3	5.16*E* − 01	−1.26		
Glycine receptor, alpha 3 [Source: HGNC Symbol; Acc: 4328]	ENST00000274093	GLRA3	8.33*E* − 02	1.64		
Glycine receptor, alpha 3, transcript variant 1	NM_006529	GLRA3	1.46*E* − 01	1.41		
Glycine receptor, alpha 4, transcript variant 1	NM_001024452	GLRA4	7.75*E* − 01	1.17		
Glycine receptor, beta, transcript variant 1	NM_000824	GLRB	6.38*E* − 02	−1.69	**6.61**	

**Table 1g:** (g) Ionotropic glutamate receptors

Gene name	Accession number	Gene symbol	P	Fold change Ker → hiPSC	Fold change hiPSC → Neuron	Fold change hiPSC → CM
Glutamate receptor, ionotropic, AMPA 1, transcript variant 1	NM_000827	GRIA1	7.65*E* − 01	−1.20	**2.06**	
Glutamate receptor, ionotropic, AMPA 2, transcript variant 1	NM_000826	GRIA2	2.87*E* − 01	−1.87	**21.26**	
Glutamate receptor, ionotropic, AMPA 2, transcript variant 1	NM_000826	GRIA2	2.72*E* − 01	1.33		
Glutamate receptor, ionotropic, AMPA 2, transcript variant 1	NM_000826	GRIA2	6.54*E* − 01	1.22		
Glutamate receptor, ionotropic, AMPA 3, transcript variant 2	NM_000828	GRIA3	5.05*E* − 01	1.26		
Glutamate receptor, ionotropic, AMPA 3 [Source: HGNC Symbol; Acc: 4573]	ENST00000371264	GRIA3	1.14*E* − 01	1.50		
Glutamate receptor, ionotropic, AMPA 3, transcript variant 2	NM_000828	GRIA3	9.05*E* − 01	−1.05		
Glutamate receptor, ionotropic, AMPA 4, transcript variant 1	NM_000829	GRIA4	2.30*E* − 02	1.80	**3.73**	
Glutamate receptor, ionotropic, delta 1	NM_017551	GRID1	1.82*E* − 01	1.50		
Glutamate receptor, ionotropic, delta 1	NM_017551	GRID1	5.08**E** − 05	**3.63**		
Glutamate receptor, ionotropic, delta 2	NM_001510	GRID2	7.64*E* − 01	−1.11		
Glutamate receptor, ionotropic, delta 2	NM_001510	GRID2	5.55**E** − 09	**55.66**		
Glutamate receptor, ionotropic, kainate 1, transcript variant 1	NM_000830	GRIK1	1.64*E* − 01	1.29	**2.58**	
Glutamate receptor, ionotropic, kainate 2, transcript variant 3	NM_001166247	GRIK2	1.42*E* − 01	1.56	**2.53**	
Glutamate receptor, ionotropic, kainate 2, transcript variant 1	NM_021956	GRIK2	1.14*E* − 01	−2.01		
Glutamate receptor, ionotropic, kainate 2, transcript variant 2	NM_175768	GRIK2	7.80*E* − 01	1.08		
Glutamate receptor, ionotropic, kainate 3	NM_000831	GRIK3	1.58*E* − 01	1.64		
Glutamate receptor, ionotropic, kainate 3	NM_000831	GRIK3	6.15**E** − 10	**22.64**		
Glutamate receptor, ionotropic, kainate 4 [Source: HGNC Symbol; Acc: 4582]	ENST00000527524	GRIK4	1.77**E** − 11	**18.10**		
Glutamate receptor, ionotropic, kainate 4	NM_014619	GRIK4	5.73*E* − 02	2.54		
Glutamate receptor, ionotropic, kainate 5	NM_002088	GRIK5	2.65*E* − 03	1.79		*−2.41 *
Glutamate receptor, ionotropic, N-methyl D-aspartate 1, transcript variant NR1-3	NM_007327	GRIN1	*1.93 E*−*02 *	*−3.02 *	**2.69**	
Glutamate receptor, ionotropic, N-methyl D-aspartate 1, transcript variant NR1-3	NM_007327	GRIN1	6.01*E* − 02	−2.45		
Glutamate receptor, ionotropic, N-methyl D-aspartate 1, transcript variant NR1-3	NM_007327	GRIN1	3.86*E* − 01	−1.65		
Glutamate receptor, ionotropic, N-methyl D-aspartate 2A, transcript variant 2	NM_000833	GRIN2A	6.54*E* − 01	1.18		
Glutamate receptor, ionotropic, N-methyl D-aspartate 2A, transcript variant 2	NM_000833	GRIN2A	9.21*E* − 01	−1.02		
Glutamate receptor, ionotropic, N-methyl D-aspartate 2A, transcript variant 1	NM_001134407	GRIN2A	3.32**E** − 10	**29.85**		
Glutamate receptor, ionotropic, N-methyl D-aspartate 2A, transcript variant 2	NM_000833	GRIN2A	2.63*E* − 01	1.55		
Glutamate receptor, ionotropic, N-methyl D-aspartate 2B	NM_000834	GRIN2B	6.64*E* − 01	−1.26		
Glutamate receptor, ionotropic, N-methyl D-aspartate 2C	NM_000835	GRIN2C	8.01*E* − 01	−1.08		
Glutamate receptor, ionotropic, N-methyl D-aspartate 2C	NM_000835	GRIN2C	5.01*E* − 01	1.13		
Glutamate receptor, ionotropic, N-methyl D-aspartate 2D	NM_000836	GRIN2D	4.69*E* − 02	−1.82		
Glutamate receptor, ionotropic, N-methyl-D-aspartate 3A	NM_133445	GRIN3A	3.28*E* − 01	−1.41		
Glutamate receptor, ionotropic, N-methyl-D-aspartate 3B	NM_138690	GRIN3B	9.36*E* − 01	−1.02		
Glutamate receptor, ionotropic, N-methyl D-aspartate-associated protein 1 (glutamate binding), transcript variant 1	NM_000837	GRINA	*3.68 E*−*04 *	*−2.05 *	**2.05**	
NMDA receptor glutamate-binding chain (hnrgw), partial	U44954	GRINA	3.06*E* − 04	−1.81		

**Table 1h:** (h) Hyperpolarization-activated cyclic nucleotide-gated channels

Gene name	Accession number	Gene symbol	P	Fold change Ker → hiPSC	Fold change hiPSC → Neuron	Fold change hiPSC → CM
Hyperpolarization-activated cyclic nucleotide-gated potassium channel 1	NM_021072	HCN1	3.47**E** − 11	**52.85**		
Hyperpolarization-activated cyclic nucleotide-gated potassium channel 1	NM_021072	HCN1	1.01**E** − 08	**9.91**		
Hyperpolarization-activated cyclic nucleotide-gated potassium channel 2	NM_001194	HCN2	5.36*E* − 02	−1.63		
Hyperpolarization-activated cyclic nucleotide-gated potassium channel 3	NM_020897	HCN3	1.98**E** − 02	**2.12**	**6.56**	
Hyperpolarization-activated cyclic nucleotide-gated potassium channel 4	NM_005477	HCN4	1.01*E* − 01	−1.66		

**Table 1i:** (i) Serotonin receptors

Gene name	Accession number	Gene symbol	*P*	Fold change Ker → hiPSC	Fold change hiPSC → Neuron	Fold change hiPSC → CM
5-hydroxytryptamine (serotonin) receptor 3A, transcript variant 1	NM_213621	HTR3A	1.99**E** − 03	**2.77**	*−2.20 *	
5-hydroxytryptamine (serotonin) receptor 3A, transcript variant 1	NM_213621	HTR3A	2.63**E** − 11	**11.11**		
5-hydroxytryptamine (serotonin) receptor 3B	NM_006028	HTR3B	1.99*E* − 01	−1.54		
5-hydroxytryptamine (serotonin) receptor 3, family member C	NM_130770	HTR3C	4.81*E* − 01	1.34		
5-hydroxytryptamine (serotonin) receptor 3 family member D, transcript variant 2	NM_182537	HTR3D	4.75*E* − 01	−1.37		
5-hydroxytryptamine (serotonin) receptor 3, family member E	NM_182589	HTR3E	1.52*E* − 01	−1.44		

**Table 1j:** (j) Voltage-gated potassium channels

Gene name	Accession number	Gene symbol	P	Fold change Ker → hiPSC	Fold change hiPSC → Neuron	Fold change hiPSC → CM
Potassium voltage-gated channel, shaker-related subfamily, member 1 (episodic ataxia with myokymia)	NM_000217	KCNA1	1.64*E* − 01	2.00		
Potassium voltage-gated channel, shaker-related subfamily, member 1 (episodic ataxia with myokymia)	NM_000217	KCNA1	1.31*E* − 01	1.87		
Potassium voltage-gated channel, shaker-related subfamily, member 2, transcript variant 2	NM_001204269	KCNA2	8.55*E* − 02	1.33		
Potassium voltage-gated channel, shaker-related subfamily, member 2, transcript variant 1	NM_004974	KCNA2	8.33*E* − 01	1.06		
Potassium voltage-gated channel, shaker-related subfamily, member 3	NM_002232	KCNA3	2.43*E* − 01	1.76		
Potassium voltage-gated channel, shaker-related subfamily, member 4	NM_002233	KCNA4	5.75*E* − 01	1.11		
Potassium voltage-gated channel, shaker-related subfamily, member 5	NM_002234	KCNA5	1.76**E** − 04	**4.11**		**5.63**
Potassium voltage-gated channel, shaker-related subfamily, member 6	NM_002235	KCNA6	7.20*E* − 02	2.28		
Potassium voltage-gated channel, shaker-related subfamily, member 7	NM_031886	KCNA7	8.53**E** − 03	**3.67**		
Potassium voltage-gated channel, shaker-related subfamily, member 7	NM_031886	KCNA7	1.04*E* − 01	2.39		
Potassium voltage-gated channel, shaker-related subfamily, member 10	NM_005549	KCNA10	3.32*E* − 01	−1.62		
Potassium voltage-gated channel, shaker-related subfamily, beta member 1, transcript variant 2	NM_003471	KCNAB1	2.70*E* − 01	1.46		
Potassium voltage-gated channel, shaker-related subfamily, beta member 1, transcript variant 2	NM_003471	KCNAB1	3.60**E** − 02	**2.42**		
Potassium voltage-gated channel, shaker-related subfamily, beta member 1, transcript variant 2	NM_003471	KCNAB1	7.87*E* − 02	3.06		
Potassium voltage-gated channel, shaker-related subfamily, beta member 2, transcript variant 1	NM_003636	KCNAB2	5.35*E* − 01	−1.35		
Potassium voltage-gated channel, shaker-related subfamily, beta member 2, transcript variant 1	NM_003636	KCNAB2	2.20*E* − 01	1.26		
Potassium voltage-gated channel, shaker-related subfamily, beta member 3 [Source: HGNC Symbol; Acc: 6230]	ENST00000303790	KCNAB3	8.14*E* − 02	1.62		
Potassium voltage-gated channel, shaker-related subfamily, beta member 3	NM_004732	KCNAB3	3.66*E* − 01	1.35		
Potassium voltage-gated channel, Shab-related subfamily, member 1	NM_004975	KCNB1	7.42**E** − 08	**16.32**	**2.59**	
Potassium voltage-gated channel, Shab-related subfamily, member 2	NM_004770	KCNB2	1.54**E** − 05	**4.58**		
Potassium voltage-gated channel, Shaw-related subfamily, member 1, transcript variant A	NM_001112741	KCNC1	1.47*E* − 01	1.75		
Potassium voltage-gated channel, Shaw-related subfamily, member 1, transcript variant B	NM_004976	KCNC1	4.86**E** − 08	**13.97**		
Potassium voltage-gated channel, Shaw-related subfamily, member 1, transcript variant A	NM_001112741	KCNC1	2.17*E* − 01	1.56		
Potassium voltage-gated channel, Shaw-related subfamily, member 2, transcript variant 1	NM_139136	KCNC2	8.53*E* − 01	1.06		
Potassium voltage-gated channel, Shaw-related subfamily, member 2, transcript variant 2	NM_139137	KCNC2	4.04*E* − 01	1.34		
Potassium voltage-gated channel, Shaw-related subfamily, member 3	NM_004977	KCNC3	3.56*E* − 01	1.20		
Potassium voltage-gated channel, Shaw-related subfamily, member 4, transcript variant 3	NM_001039574	KCNC4	7.70*E* − 02	−1.40		
Potassium voltage-gated channel, Shal-related subfamily, member 1	NM_004979	KCND1	1.48**E** − 04	**7.11**		
Potassium voltage-gated channel, Shal-related subfamily, member 2	NM_012281	KCND2	1.62**E** − 12	**91.08**	**2.76**	*−2.57 *
Potassium voltage-gated channel, Shal-related subfamily, member 2	NM_012281	KCND2	2.76**E** − 09	**21.83**		
Potassium voltage-gated channel, Shal-related subfamily, member 3 [Source: HGNC Symbol; Acc: 6239]	ENST00000369697	KCND3	5.36*E* − 01	−1.36		
Potassium voltage-gated channel, Shal-related subfamily, member 3, transcript variant 1	NM_004980	KCND3	1.05*E* − 01	1.55		
Potassium voltage-gated channel, Shal-related subfamily, member 3, transcript variant 1	NM_004980	KCND3	4.41*E* − 01	1.37		
Potassium voltage-gated channel, Shal-related subfamily, member 3 [Source: HGNC Symbol; Acc: 6239]	ENST00000369697	KCND3	3.86*E* − 04	−1.84		
Potassium voltage-gated channel, Isk-related family, member 1, transcript variant 2	NM_000219	KCNE1	3.53*E* − 01	−1.45		
Potassium voltage-gated channel, Isk-related family, member 1, transcript variant 2	NM_000219	KCNE1	3.59*E* − 01	1.25		
KCNE1-like	NM_012282	KCNE1L	1.34**E** − 08	**4.44**		*−2.18 *
KCNE1-like	NM_012282	KCNE1L	1.11**E** − 08	**3.65**		
KCNE1-like	NM_012282	KCNE1L	6.41*E* − 03	1.88		
Potassium voltage-gated channel, Isk-related family, member 2	NM_172201	KCNE2	1.41**E** − 04	**3.93**		
Potassium voltage-gated channel, Isk-related family, member 3	NM_005472	KCNE3	8.65**E** − 15	**52.36**		
Potassium voltage-gated channel, Isk-related family, member 4	NM_080671	KCNE4	5.04*E* − 01	1.20		
Potassium voltage-gated channel, subfamily F, member 1	NM_002236	KCNF1	2.25**E** − 05	**6.48**	**2.84**	
Potassium voltage-gated channel, subfamily G, member 1	NM_002237	KCNG1	8.51*E* − 03	−1.82		
Potassium voltage-gated channel, subfamily G, member 1	NM_002237	KCNG1	1.55*E* − 01	−1.65		
Potassium voltage-gated channel, subfamily G, member 2	NM_012283	KCNG2	6.21*E* − 02	1.32		
Potassium voltage-gated channel, subfamily G, member 3, transcript variant 1	NM_133329	KCNG3	3.74**E** − 11	**58.95**		*−2.93 *
Potassium voltage-gated channel, subfamily G, member 4	NM_172347	KCNG4	*3.36 E*−*03 *	*−3.33 *		
Potassium voltage-gated channel, subfamily G, member 4, (cDNA clone IMAGE: 3028985)	BC008969	KCNG4	*2.24 E*−*04 *	*−3.29 *		
Potassium voltage-gated channel, subfamily H (eag-related), member 1, transcript variant 1	NM_172362	KCNH1	8.05*E* − 01	−1.15		
Potassium voltage-gated channel, subfamily H (eag-related), member 2, transcript variant 1	NM_000238	KCNH2	5.94**E** − 04	**37.53**		
Potassium voltage-gated channel, subfamily H (eag-related), member 2, transcript variant 2	NM_172056	KCNH2	5.70*E* − 03	1.73		
Potassium voltage-gated channel, subfamily H (eag-related), member 3	NM_012284	KCNH3	9.65*E* − 01	1.01		
Potassium voltage-gated channel, subfamily H (eag-related), member 4	NM_012285	KCNH4	3.18*E* − 01	−1.24		
Potassium voltage-gated channel, subfamily H (eag-related), member 5, transcript variant 2	NM_172376	KCNH5	3.04**E** − 03	**2.96**		
Potassium voltage-gated channel, subfamily H (eag-related), member 5, transcript variant 1	NM_139318	KCNH5	5.41*E* − 01	1.47		
Potassium voltage-gated channel, subfamily H (eag-related), member 6, transcript variant 2	NM_173092	KCNH6	2.93**E** − 02	**3.04**		
cDNA FLJ33650 fis, clone BRAMY2024514, highly similar to Rattus norvegicus Potassium channel (erg2)	AK090969	KCNH6	2.08**E** − 05	**12.42**		
Potassium voltage-gated channel, subfamily H (eag-related), member 6, transcript variant 2	NM_173092	KCNH6	2.55**E** − 02	**2.29**		
Potassium voltage-gated channel, subfamily H (eag-related), member 7, transcript variant 2	NM_173162	KCNH7	4.63*E* − 01	−1.40		
Potassium voltage-gated channel, subfamily H (eag-related), member 7, transcript variant 1	NM_033272	KCNH7	7.58*E* − 01	−1.09		
Potassium voltage-gated channel, subfamily H (eag-related), member 8	NM_144633	KCNH8	3.00**E** − 09	**24.57**	**2.97**	
Kv channel interacting protein 1, transcript variant 1	NM_001034837	KCNIP1	8.33*E* − 02	1.96	**4.24**	
Kv channel interacting protein 2, transcript variant 7	NM_173197	KCNIP2	6.43*E* − 01	1.21		
Kv channel interacting protein 2, transcript variant 1	NM_014591	KCNIP2	2.53*E* − 03	1.96		
Kv channel interacting protein 3, calsenilin, transcript variant 1	NM_013434	KCNIP3	*1.15 E*−*03 *	*−3.47 *		
Kv channel interacting protein 4, transcript variant 5	NM_001035003	KCNIP4	6.73*E* − 01	1.16		**2.01**
Kv channel interacting protein 4, transcript variant 5	NM_001035003	KCNIP4	9.26*E* − 01	1.05		
Potassium voltage-gated channel, KQT-like subfamily, member 1, transcript variant 1	NM_000218	KCNQ1	3.31**E** − 09	**79.52**		
Potassium voltage-gated channel, KQT-like subfamily, member 2, transcript variant 5	NM_172109	KCNQ2	5.68**E** − 19	**3408.43**		
Potassium voltage-gated channel, KQT-like subfamily, member 2, transcript variant 3	NM_004518	KCNQ2	1.29*E* − 02	1.82		
Potassium voltage-gated channel, KQT-like subfamily, member 2, (cDNA clone IMAGE: 4154700)	BC020384	KCNQ2	1.09**E** − 08	**37.12**		
Potassium voltage-gated channel, KQT-like subfamily, member 3, transcript variant 1	NM_004519	KCNQ3	1.10*E* − 04	−1.69		
Potassium voltage-gated channel, KQT-like subfamily, member 4, transcript variant 1	NM_004700	KCNQ4	9.77*E* − 01	1.02		
Potassium voltage-gated channel, KQT-like subfamily, member 5, transcript variant 1	NM_019842	KCNQ5	9.81*E* − 02	−1.79		
Potassium voltage-gated channel, delayed-rectifier, subfamily S, member 1	NM_002251	KCNS1	3.39*E* − 01	−1.27		*−2.64 *
Potassium voltage-gated channel, delayed-rectifier, subfamily S, member 2	NM_020697	KCNS2	6.19*E* − 02	−2.45	**2.11**	
Potassium voltage-gated channel, delayed-rectifier, subfamily S, member 3	NM_002252	KCNS3	1.04**E** − 04	**2.22**	*−2.61 *	*−2.39 *
Potassium channel, subfamily T, member 1 (sodium activated)	NM_020822	KCNT1	8.51*E* − 02	−2.17		
Potassium channel, subfamily V, member 1	NM_014379	KCNV1	7.84*E* − 02	1.58		
Potassium channel, subfamily V, member 2	NM_133497	KCNV2	7.63*E* − 01	−1.16		

**Table 1k:** (k) Inwardly rectifying potassium channels

Gene name	Accession number	Gene symbol	P	Fold change Ker → hiPSC	Fold change hiPSC → Neuron	Fold change hiPSC → CM
Potassium inwardly-rectifying channel, subfamily J, member 1, transcript variant rom-k5	NM_153767	KCNJ1	2.54**E** − 02	**2.66**		
Potassium inwardly-rectifying channel, subfamily J, member 1, transcript variant rom-k5	NM_153767	KCNJ1	8.51*E* − 01	−1.05		
Potassium inwardly-rectifying channel, subfamily J, member 2	NM_000891	KCNJ2	5.14**E** − 04	**4.38**		
Potassium inwardly-rectifying channel, subfamily J, member 3	NM_002239	KCNJ3	4.56*E* − 01	1.34		
Potassium inwardly-rectifying channel, subfamily J, member 4, transcript variant 1	NM_152868	KCNJ4	4.80**E** − 02	**2.30**		
Potassium inwardly-rectifying channel, subfamily J, member 5	NM_000890	KCNJ5	*1.88 E*−*08 *	*−45.40 *		
Potassium inwardly-rectifying channel, subfamily J, member 5	NM_000890	KCNJ5	*4.42 E*−*12 *	*−109.11 *		
Potassium inwardly-rectifying channel, subfamily J, member 6	NM_002240	KCNJ6	3.29**E** − 05	**6.75**	**5.54**	
Potassium inwardly-rectifying channel, subfamily J, member 8	NM_004982	KCNJ8	1.98**E** − 04	**5.97**		**2.74**
Potassium inwardly-rectifying channel, subfamily J, member 9 [Source: HGNC Symbol; Acc: 6270]	ENST00000368088	KCNJ9	3.17*E* − 01	1.49		
Potassium inwardly-rectifying channel, subfamily J, member 9	NM_004983	KCNJ9	3.07*E* − 01	1.79		
Potassium inwardly-rectifying channel, subfamily J, member 10	NM_002241	KCNJ10	8.85**E** − 04	**4.14**		
Potassium inwardly-rectifying channel, subfamily J, member 10	NM_002241	KCNJ10	9.56*E* − 01	1.01		
Potassium inwardly-rectifying channel, subfamily J, member 11, transcript variant 1	NM_000525	KCNJ11	4.00**E** − 04	**2.93**		
Potassium inwardly-rectifying channel, subfamily J, member 12	NM_021012	KCNJ12	1.00*E* − 01	1.47		
Potassium inwardly-rectifying channel, subfamily J, member 12	NM_021012	KCNJ12	3.53**E** − 06	**5.81**		
Potassium inwardly-rectifying channel, subfamily J, member 13, transcript variant 1	NM_002242	KCNJ13	7.75*E* − 01	1.20		
Potassium inwardly-rectifying channel, subfamily J, member 14, transcript variant 2	NM_170720	KCNJ14	9.05*E* − 02	−1.40		
Potassium inwardly-rectifying channel, subfamily J, member 15, transcript variant 1	NM_170736	KCNJ15	*3.41 E*−*08 *	*−150.95 *		
Potassium inwardly-rectifying channel, subfamily J, member 16, transcript variant 2	NM_170741	KCNJ16	2.22*E* − 01	−1.93		

**Table 1l:** (l) Two-P potassium channels

Gene name	Accession number	Gene symbol	P	Fold change Ker → hiPSC	Fold change hiPSC → Neuron	Fold change hiPSC → CM
Potassium channel, subfamily K, member 1	NM_002245	KCNK1	3.02*E* − 04	−1.68		
Potassium channel, subfamily K, member 2, transcript variant 1	NM_001017424	KCNK2	3.46*E* − 01	1.57		
Potassium channel, subfamily K, member 3	NM_002246	KCNK3	9.98*E* − 01	1.00	**2.36**	
Potassium channel, subfamily K, member 3	NM_002246	KCNK3	8.75*E* − 01	−1.10		
Potassium channel, subfamily K, member 3	NM_002246	KCNK3	5.34*E* − 01	−1.30		
Potassium channel, subfamily K, member 3 [Source: HGNC Symbol; Acc: 6278]	ENST00000302909	KCNK3	2.53*E* − 01	1.52		
Potassium channel, subfamily K, member 4	NM_033310	KCNK4	6.21*E* − 01	−1.14	**2.05**	
Potassium channel, subfamily K, member 5	NM_003740	KCNK5	1.16**E** − 14	**29.46**	*−2.35 *	*−2.03 *
Potassium channel, subfamily K, member 6	NM_004823	KCNK6	*1.22 E*−*06 *	*−3.48 *	*−2.20 *	
Potassium channel, subfamily K, member 6	NM_004823	KCNK6	*2.05 E*−*08 *	*−5.11 *		
Potassium channel, subfamily K, member 6	NM_004823	KCNK6	*7.04E−06 *	*−5.22 *		
Potassium channel, subfamily K, member 7, transcript variant A	NM_033347	KCNK7	1.17*E* − 01	−1.52		
Potassium channel, subfamily K, member 9	NM_016601	KCNK9	6.67*E* − 01	−1.29		
Potassium channel, subfamily K, member 10, transcript variant 1	NM_021161	KCNK10	2.63*E* − 01	−1.30		
Potassium channel, subfamily K, member 10, transcript variant 2	NM_138317	KCNK10	1.41*E* − 02	1.43		
Potassium channel, subfamily K, member 12	NM_022055	KCNK12	4.58**E** − 10	**23.40**		*−5.58 *
Potassium channel, subfamily K, member 13	NM_022054	KCNK13	7.65*E* − 01	−1.10		
Potassium channel, subfamily K, member 15	NM_022358	KCNK15	4.96*E* − 02	−1.50		
Potassium channel, subfamily K, member 15	NM_022358	KCNK15	9.10*E* − 01	−1.09		
Pancreatic potassium channel TALK-1d; alternatively spliced	AY253147	KCNK16	*2.17 E*−*02 *	*−2.43 *		
Potassium channel, subfamily K, member 17, transcript variant 1	NM_031460	KCNK17	1.87**E** − 11	**28.60**		
Potassium channel, subfamily K, member 17, transcript variant 1	NM_031460	KCNK17	1.52**E** − 05	**19.62**		
Potassium channel, subfamily K, member 18	NM_181840	KCNK18	1.77*E* − 01	1.61		

**Table 1m:** (m) Calcium-activated potassium channels

Gene name	Accession number	Gene symbol	P	Fold change Ker → hiPSC	Fold change hiPSC → Neuron	Fold change hiPSC → CM
Potassium large conductance calcium-activated channel, subfamily M, alpha member 1, transcript variant 2	NM_002247	KCNMA1	1.01*E* − 02	1.84		**2.01**
Potassium large conductance calcium-activated channel, subfamily M, alpha member 1, transcript variant 1	NM_001014797	KCNMA1	3.19*E* − 01	1.32		
Potassium large conductance calcium-activated channel, subfamily M, alpha member 1, transcript variant 2	NM_002247	KCNMA1	3.67**E** − 03	**2.27**		
Potassium large conductance calcium-activated channel, subfamily M, alpha member 1, transcript variant 2	NM_002247	KCNMA1	8.53*E* − 02	1.44		
Maxi-K channel HSLO	AF349445	KCNMA1	3.04*E* − 01	1.44		
Potassium large conductance calcium-activated channel, subfamily M, alpha member 1, transcript variant 2	NM_002247	KCNMA1	2.15*E* − 01	−1.23	
Potassium large conductance calcium-activated channel, subfamily M, beta member 1	NM_004137	KCNMB1	7.12*E* − 02	−1.41		
Potassium large conductance calcium-activated channel, subfamily M, beta member 1	NM_004137	KCNMB1	2.23**E** − 06	**7.82**		
Potassium large conductance calcium-activated channel, subfamily M, beta member 2, transcript variant 1	NM_181361	KCNMB2	3.36*E* − 01	1.56		
Potassium large conductance calcium-activated channel, subfamily M, beta member 2, transcript variant 1	NM_181361	KCNMB2	1.17*E* − 01	1.47		
Potassium large conductance calcium-activated channel, subfamily M beta member 3, transcript variant 1	NM_171828	KCNMB3	1.41**E** − 05	**2.63**		
Potassium large conductance calcium-activated channel, subfamily M, beta member 4	NM_014505	KCNMB4	8.85**E** − 12	**103.99**		
Potassium large conductance calcium-activated channel, subfamily M, beta member 4 [Source: HGNC Symbol; Acc: 6289]	ENST00000258111	KCNMB4	9.22**E** − 10	**18.40**		
Potassium intermediate/small conductance calcium-activated channel, subfamily N, member 1	NM_002248	KCNN1	3.40**E** − 05	**4.67**		
Potassium intermediate/small conductance calcium-activated channel, subfamily N, member 2, transcript variant 1	NM_021614	KCNN2	1.88**E** − 13	**22.23**	*−3.63 *	
Potassium intermediate/small conductance calcium-activated channel, subfamily N, member 3, transcript variant 1	NM_002249	KCNN3	2.58**E** − 06	**8.25**		
Potassium intermediate/small conductance calcium-activated channel, subfamily N, member 3, transcript variant 3	NM_001204087	KCNN3	1.11**E** − 07	**15.29**		
Potassium intermediate/small conductance calcium-activated channel, subfamily N, member 4	NM_002250	KCNN4	1.06*E* − 02	−1.84		

**Table 1n:** (n) P2X receptors

Gene name	Accession number	Gene symbol	P	Fold change Ker → hiPSC	Fold change hiPSC → Neuron	Fold change hiPSC → CM
Purinergic receptor P2X, ligand-gated ion channel, 1	NM_002558	P2RX1	7.71*E* − 01	1.14		
Purinergic receptor P2X, ligand-gated ion channel, 2, transcript variant 4	NM_170683	P2RX2	5.25*E* − 02	−1.66		
Purinergic receptor P2X, ligand-gated ion channel, 3	NM_002559	P2RX3	2.43*E* − 02	−1.76		
Purinergic receptor P2X, ligand-gated ion channel, 4	NM_002560	P2RX4	8.68*E* − 01	−1.03		
Purinergic receptor P2X, ligand-gated ion channel, 5, transcript variant 1	NM_002561	P2RX5	7.68**E** − 08	**6.97**		
Purinergic receptor P2X, ligand-gated ion channel, 5, transcript variant 2	NM_175080	P2RX5	6.13*E* − 02	1.54		
Purinergic receptor P2X, ligand-gated ion channel, 6 [Source: HGNC Symbol; Acc: 8538]	ENST00000413302	P2RX6	4.13*E* − 01	1.68		
Purinergic receptor P2X, ligand-gated ion channel, 7, transcript variant 1	NM_002562	P2RX7	6.13*E* − 01	1.23		
Purinergic receptor P2X, ligand-gated ion channel, 7, transcript variant 1	NM_002562	P2RX7	9.17*E* − 01	1.04		

**Table 1o:** (o) Transient receptor potential channels

Gene name	Accession number	Gene symbol	P	Fold change Ker → hiPSC	Fold change hiPSC → Neuron	Fold change hiPSC → CM
Polycystic kidney disease 2 (autosomal dominant)	NM_000297	PKD2	7.48*E* − 07	1.79		
Polycystic kidney disease 2 (autosomal dominant)	NM_000297	PKD2	2.38*E* − 05	1.98		
Polycystic kidney disease 2-like 1	NM_016112	PKD2L1	1.29**E** − 03	**4.66**		
Polycystic kidney disease 2-like 2	NM_014386	PKD2L2	1.94*E* − 01	1.49		
Polycystic kidney disease 2-like 2	NM_014386	PKD2L2	7.43*E* − 01	1.19		
Polycystic kidney disease 2-like 2	NM_014386	PKD2L2	6.74*E* − 01	1.09		
Transient receptor potential cation channel, subfamily A, member 1	NM_007332	TRPA1	2.99*E* − 01	1.74		
Transient receptor potential cation channel, subfamily A, member 1	NM_007332	TRPA1	5.64*E* − 02	2.70		
Transient receptor potential cation channel, subfamily C, member 1	NM_003304	TRPC1	4.30**E** − 06	**2.01**		
Transient receptor potential cation channel, subfamily C, member 3, transcript variant 2	NM_003305	TRPC3	1.12**E** − 07	**5.78**		**2.45**
Transient receptor potential cation channel, subfamily C, member 4, transcript variant alpha	NM_016179	TRPC4	3.25**E** − 07	**25.38**		
Transient receptor potential cation channel, subfamily C, member 5	NM_012471	TRPC5	1.20*E* − 01	1.28		
Transient receptor potential cation channel, subfamily C, member 6	NM_004621	TRPC6	1.21*E* − 01	2.33		
Transient receptor potential cation channel, subfamily C, member 7, transcript variant 1	NM_020389	TRPC7	6.18*E* − 01	−1.36		
Transient receptor potential cation channel, subfamily M, member 1	NM_002420	TRPM1	2.29*E* − 01	1.31		
Transient receptor potential cation channel, subfamily M, member 2, transcript variant 1	NM_003307	TRPM2	1.22*E* − 01	1.97		
Transient receptor potential cation channel, subfamily M, member 2, transcript variant 1	NM_003307	TRPM2	5.04*E* − 02	2.53		
Transient receptor potential cation channel, subfamily M, member 3, transcript variant 7	NM_206948	TRPM3	3.04**E** − 09	**10.69**		
Transient receptor potential cation channel, subfamily M, member 3 [Source: HGNC Symbol; Acc: 17992]	ENST00000354500	TRPM3	2.34**E** − 03	**3.74**		
Transient receptor potential cation channel, subfamily M, member 3, transcript variant 7	NM_206948	TRPM3	3.41**E** − 06	**6.23**		
Transient receptor potential cation channel, subfamily M, member 3, transcript variant 9	NM_001007471	TRPM3	7.23*E* − 01	1.13		
Transient receptor potential cation channel, subfamily M, member 3, transcript variant 9	NM_001007471	TRPM3	1.54**E** − 05	**2.60**		
Transient receptor potential cation channel, subfamily M, member 4, transcript variant 1	NM_017636	TRPM4	3.72*E* − 02	−1.89		
Transient receptor potential cation channel, subfamily M, member 5	NM_014555	TRPM5	8.21*E* − 01	−1.08		
Transient receptor potential cation channel, subfamily M, member 6, transcript variant a	NM_017662	TRPM6	3.80*E* − 01	1.64		
Transient receptor potential cation channel, subfamily M, member 6, transcript variant a	NM_017662	TRPM6	9.10*E* − 01	1.05		
Transient receptor potential cation channel, subfamily M, member 6, transcript variant a	NM_017662	TRPM6	5.32**E** − 07	**5.59**		
Transient receptor potential cation channel, subfamily M, member 6, transcript variant a	NM_017662	TRPM6	3.32*E* − 01	1.36		
Transient receptor potential cation channel, subfamily M, member 7	NM_017672	TRPM7	2.16*E* − 02	−1.25		
Transient receptor potential cation channel, subfamily M, member 7	NM_017672	TRPM7	3.64*E* − 03	−1.38		
Transient receptor potential cation channel, subfamily M, member 8	NM_024080	TRPM8	2.04*E* − 01	1.89		
Transient receptor potential cation channel, subfamily V, member 1, transcript variant 3	NM_080706	TRPV1	3.59*E* − 05	1.92		
Transient receptor potential cation channel, subfamily V, member 1, transcript variant 3	NM_080706	TRPV1	4.14**E** − 05	**2.25**		
Transient receptor potential cation channel, subfamily V, member 2	NM_016113	TRPV2	8.02**E** − 08	**4.68**		
Transient receptor potential cation channel, subfamily V, member 3	NM_145068	TRPV3	4.64*E* − 01	1.35		
Transient receptor potential cation channel, subfamily V, member 3	NM_145068	TRPV3	1.35*E* − 01	−2.39		
Transient receptor potential cation channel, subfamily V, member 4, transcript variant 2	NM_147204	TRPV4	4.33*E* − 01	1.39		
Transient receptor potential cation channel, subfamily V, member 5, (cDNA clone MGC: 34269 IMAGE: 5186668)	BC034740	TRPV5	1.00*E* − 01	1.38		
Transient receptor potential cation channel, subfamily V, member 5	NM_019841	TRPV5	3.14*E* − 01	−1.40		
Transient receptor potential cation channel, subfamily V, member 6	NM_018646	TRPV6	1.57*E* − 01	−1.38		

**Table 1p:** (p) Voltage-gated sodium channels

Gene name	Accession number	Gene symbol	P	Fold change Ker → hiPSC	Fold change hiPSC → Neuron	Fold change hiPSC → CM
Sodium channel, voltage-gated, type I, alpha subunit, transcript variant 2	NM_006920	SCN1A	1.98*E* − 01	1.54		
Sodium channel, voltage-gated, type I, beta, transcript variant b	NM_199037	SCN1B	9.95*E* − 01	−1.00		
Sodium channel, voltage-gated, type II, alpha subunit, transcript variant 1	NM_021007	SCN2A	4.16*E* − 01	1.62	**5.00**	
Sodium channel, voltage-gated, type II, beta	NM_004588	SCN2B	3.61*E* − 01	−1.58		
Sodium channel, voltage-gated, type II, beta	NM_004588	SCN2B	*8.16 E*−*04 *	*−2.41 *		
Sodium channel, voltage-gated, type III, alpha subunit, transcript variant 1	NM_006922	SCN3A	2.87*E* − 01	−2.00	**2.85**	
Sodium channel, voltage-gated, type III, alpha subunit, transcript variant 1	NM_006922	SCN3A	1.89*E* − 01	1.49		
Sodium channel, voltage-gated, type III, beta, transcript variant 1	NM_018400	SCN3B	8.74*E* − 01	−1.06	**4.70**	
Sodium channel, voltage-gated, type III, beta, transcript variant 1	NM_018400	SCN3B	1.57*E* − 01	−1.46		
Sodium channel, voltage-gated, type III, beta, transcript variant 1	NM_018400	SCN3B	1.56*E* − 01	1.96		
Sodium channel, voltage-gated, type IV, alpha subunit	NM_000334	SCN4A	1.62**E** − 08	**9.91**		
Sodium channel, voltage-gated, type IV, beta, transcript variant 1	NM_174934	SCN4B	*3.25 E*−*04 *	*−3.66 *		
Sodium channel, voltage-gated, type V, alpha subunit, transcript variant 1	NM_198056	SCN5A	2.43**E** − 08	**14.10**		
Sodium channel, voltage-gated, type V, alpha subunit, transcript variant 2	NM_000335	SCN5A	4.93*E* − 02	1.95		
Sodium channel, voltage-gated, type VII, alpha	NM_002976	SCN7A	4.49*E* − 03	1.63		
Sodium channel, voltage-gated, type VII, alpha	NM_002976	SCN7A	2.07*E* − 01	1.72		
Sodium channel, voltage gated, type VIII, alpha subunit, transcript variant 1	NM_014191	SCN8A	1.89**E** − 09	**15.61**		
Sodium channel, voltage gated, type VIII, alpha subunit, transcript variant 1	NM_014191	SCN8A	4.06**E** − 07	**11.23**		
Sodium channel, voltage-gated, type IX, alpha subunit	NM_002977	SCN9A	4.08**E** − 08	**11.51**		
Sodium channel, voltage-gated, type IX, alpha subunit	NM_002977	SCN9A	1.62*E* − 01	1.66		
Sodium channel, voltage-gated, type IX, alpha subunit	NM_002977	SCN9A	3.38**E** − 03	**2.57**		
Sodium channel, voltage-gated, type X, alpha subunit	NM_006514	SCN10A	4.06*E* − 01	1.35		
Sodium channel, voltage-gated, type XI, alpha subunit	NM_014139	SCN11A	3.02*E* − 01	1.57		
Sodium channel, voltage-gated, type XI, alpha subunit [Source: HGNC Symbol; Acc: 10583]	ENST00000444237	SCN11A	8.83*E* − 03	1.94		

**Table 1q:** (q) Nonvoltage-gated sodium channels

Gene name	Accession number	Gene symbol	P	Fold change Ker → hiPSC	Fold change hiPSC → Neuron	Fold change hiPSC → CM
Sodium channel, nonvoltage-gated 1 alpha, transcript variant 1	NM_001038	SCNN1A	1.33**E** − 09	**51.49**	*−21.13 *	*−10.71 *
Sodium channel, nonvoltage-gated 1, beta	NM_000336	SCNN1B	2.11*E* − 01	1.64		
Sodium channel, nonvoltage-gated 1, beta	NM_000336	SCNN1B	5.52*E* − 01	1.09		
Sodium channel, nonvoltage-gated 1, delta, transcript variant 1	NM_001130413	SCNN1D	8.43*E* − 02	1.49		
Sodium channel, nonvoltage-gated 1, gamma	NM_001039	SCNN1G	2.33*E* − 01	1.59		
Gamma subunit of epithelial amiloride-sensitive sodium channel	X87160	SCNN1G	3.99*E* − 01	1.27		

**Table 1r:** (r) Two-pore channels

Gene name	Accession number	Gene symbol	P	Fold change Ker → hiPSC	Fold change hiPSC → Neuron	Fold change hiPSC → CM
Two-pore segment channel 1, transcript variant 1	NM_001143819	TPCN1	6.20*E* − 04	−1.76		
Two-pore segment channel 1, transcript variant 2	NM_017901	TPCN1	*1.71 E*−*03 *	*−2.10 *		
Two-pore segment channel 1, transcript variant 2	NM_017901	TPCN1	*3.55 E*−*04 *	*−2.44 *		
Two-pore segment channel 1, transcript variant 1	NM_001143819	TPCN1	8.06*E* − 03	−1.83		
Two-pore segment channel 2	NM_139075	TPCN2	5.06*E* − 01	−1.15		

**Table 1s:** (s) Zinc-activated ligand-gated ion channels

Gene name	Accession number	Gene symbol	P	Fold change Ker → hiPSC	Fold change hiPSC → Neuron	Fold change hiPSC → CM
Zinc-activated ligand-gated ion channel	NM_180990	ZACN	9.24*E* − 02	1.45		
